# Surgeon’s experience, sports participation and a concomitant MCL injury increase the use of patellar and quadriceps tendon grafts in primary ACL reconstruction: a nationwide registry study of 39,964 surgeries

**DOI:** 10.1007/s00167-022-07057-5

**Published:** 2022-07-27

**Authors:** Dzan Rizvanovic, Markus Waldén, Magnus Forssblad, Anders Stålman

**Affiliations:** 1 Department of Molecular Medicine and Surgery, Stockholm Sports Trauma Research Center, Karolinska Institutet, Stockholm, Sweden; 2 Department of Orthopaedics, Region Kronoberg, Växjö, Sweden; 3 Unit of Public Health, Department of Health, Medicine and Caring Sciences, Linköping University, Linköping, Sweden; 4 GHP Ortho & Spine Center Skåne, Malmö, Sweden; 5 Ortopedi Stockholm, Stockholm, Sweden; 6 Capio Artro Clinic, Sophiahemmet, Stockholm, Sweden

**Keywords:** Caseload, Concomitant injuries, Hamstring, Knee, Ligament reconstruction, Operating volume, Patellar, Quadriceps, Surgical technique

## Abstract

**Purpose:**

To investigate the influence of surgeon-related factors and clinic routines on autograft choice in primary anterior cruciate ligament reconstruction (ACLR).

**Methods:**

Data from the Swedish National Knee Ligament Registry (SNKLR), 2008–2019, were used to study autograft choice (hamstring; HT, patellar; PT, or quadriceps tendon; QT) in primary ACLR. Patient/injury characteristics (sex, age at surgery, activity at time of injury and associated injuries) and surgeon-/clinic-related factors (operating volume, caseload and graft type use) were analyzed. Surgeon/clinic volume was divided into tertiles (low-, mid- and high-volume categories). Multivariable logistic regression was performed to assess variables influencing autograft choice in 2015–2019, presented as the odds ratio (OR) with a 95% confidence interval (CI).

**Results:**

39,964 primary ACLRs performed by 299 knee surgeons in 91 clinics were included. Most patients received HT (93.7%), followed by PT (4.2%) and QT (2.1%) grafts. Patients were mostly operated on by high-volume (> 28 ACLRs/year) surgeons (68.1%), surgeons with a caseload of ≥ 50 ACLRs (85.1%) and surgeons with the ability to use ≥ two autograft types (85.9%) (all *p* < 0.001). Most patients underwent ACLR at high-volume (> 55 ACLRs/year) clinics (72.2%) and at clinics capable of using ≥ two autograft types (93.1%) (both *p* < 0.001). Significantly increased odds of receiving PT/QT autografts were found for ACLR by surgeons with a caseload of ≥ 50 ACLRs (OR 1.41, 95% CI 1.11–1.79), but also for injury during handball (OR 1.31, 95% CI 1.02–1.67), various other pivoting sports (basketball, hockey, rugby and American football) (OR 1.59, 95% CI 1.24–2.03) and a concomitant medial collateral ligament (MCL) injury (OR 4.93, 95% CI 4.18–5.80). In contrast, female sex (OR 0.87, 95% CI 0.77–0.97), injury during floorball (OR 0.71, 95% CI 0.55–0.91) and ACLR by mid-volume relative to high-volume surgeons (OR 0.62, 95% CI 0.53–0.73) had significantly reduced odds of receiving PT/QT autografts.

**Conclusion:**

An HT autograft was used in the vast majority of cases, but PT/QT autografts were used more frequently by experienced surgeons. Prior research has demonstrated significant differences in autograft characteristics. For this reason, patients might benefit if surgery is performed by more experienced surgeons.

**Level of evidence:**

Level III.

## Introduction

Anterior cruciate ligament (ACL) rupture is a serious knee injury, often affecting young and active individuals [30, 48]. Approximately 42.5 ACL surgeries including revisions are performed per 100,000 people in Sweden [48]. This is similar to previous estimates from the United States [26]. Primary ACL reconstruction (ACLR) is mostly performed with hamstring tendon (HT) or patellar tendon (PT) autografts [32, 48, 52], but the quadriceps tendon (QT) autograft has increased in popularity in recent years [41, 48].

Autograft selection in primary ACLR still remains controversial. Previous studies indicate lower surgical morbidity for the HT graft [17, 19, 54], but improved stability [6, 19] and a reduced risk of re-rupture/revision for the PT graft [7, 8, 20, 27, 28, 37]. The QT graft is less well studied, but findings suggest that the QT graft produces less donor-site morbidity and the same stability and risk of re-rupture as the PT graft [29].

Several studies highlight the importance of an individualized approach regarding treatment and graft selection based on patient characteristics and desired activity level [9, 16, 20, 28, 37, 53]. However, the main factor influencing the graft choice decision is often the surgeon’s preference [28, 36]. The surgeon’s preference in terms of graft selection depends on many factors, some of them with little or no evidence, such as surgeon’s experience and clinic routines [2, 4].

The aim of this nationwide registry-based study was to investigate the influence of surgeon-related factors and clinic routines on autograft choice. It was hypothesized that patients would receive more PT and QT autografts if operated on by more experienced surgeons and at high-volume clinics.

## Materials and methods

The Swedish National Knee Ligament Registry (SNKLR) was established in January 2005 and covers more than 90% of all the ACLRs performed annually in Sweden. By the end of 2019, the registry included more than 49,000 primary ACLRs and around 3700 revision procedures [48]. Surgeons report patient and injury characteristics, concomitant knee injuries, intraoperative findings, surgical procedures, graft choice, implant selection, any immediate perioperative complications and duration of the surgery using a standardized web-based protocol. All patients are asked to fill in patient-reported outcomes in the register pre- and postoperatively. Patient demographics (age and sex) are collected through Swedish social security numbers. Any subsequent ACL revision surgery and contralateral ACLR is reported separately and is linked to the index surgery. Participation in the SNKLR is voluntary for both patients and surgeons. Ethical approval was obtained from the Regional Ethical Review Board in Stockholm (2011/337-31/3). To conduct a study based on data from the SNKLR, subsequent approval from the steering committee of the SNKLR is also required (granted 2020-05-20).

### Study design and patient selection

A retrospective registry-based cohort study was conducted to determine autograft choice in primary ACLR and to examine patient-, injury-, surgeon- and clinic-related factors that might influence graft selection. Patients registered for an ACLR in the SNKLR between January 1, 2006, and December 31, 2019, were assessed for eligibility. The total number of ACLRs prior to the start of the SNKLR is unknown and, for this reason, patients undergoing surgery in 2006–2007 were excluded, since it was impossible to analyze the actual surgeon experience during the first registered years. The data registered in the SNKLR from 2006 and 2007 have only been taken into account regarding the number of consecutive procedures, since this was needed for the registry caseload calculations. Additionally, ACL revision surgery and primary ACLRs with associated injuries to the lateral collateral ligament (LCL), posterior cruciate ligament (PCL) and posterolateral complex (PLC) were excluded from all analyses, as well as concomitant fractures requiring specific fracture treatment (except for intra-articular impressions and Segond fractures) and injuries to tendons, nerves and major blood vessels. Finally, patients were excluded if they received a graft other than HT, PT or QT autografts and if the surgeon code was missing.

### Outcomes

The primary outcome measurements of interest were surgeon- and clinic-related factors, as specified in detail below, and their association with the selection of HT, PT or QT autografts. The secondary outcomes were various patient- and injury-related variables.

#### Patient and injury characteristics

The following patient- and injury-related factors were studied: sex, age at surgery, activity at the time of injury (pivoting or non-pivoting), concomitant medial collateral ligament (MCL) injury at surgery, associated meniscal or cartilage injury at surgery and year of surgery. Pivoting activities were defined as football, floorball, handball and other sports (basketball, hockey, rugby and American football — grouped together due to low participation rates). Non-pivoting activities were other sports, exercise, work, traffic accident or any other specified reasons.

#### Surgeon-related factors

Surgeon-related factors included surgeon volume (annual number of ACLRs/revisions per surgeon during the calendar year when the primary ACLR was performed), average surgeon volume (median number of ACLRs/revisions per surgeon during the three most active years), surgeon caseload (primary ACLRs performed by a surgeon with a registry caseload of ≥ 50 ACLRs/revisions) and the surgeon’s ability to use different graft types (HT, PT and QT). All registered ACLRs and revisions in the SNKLR between 2008 and 2019 were assessed to illustrate the surgeon’s operating volume.

#### Clinic-related factors

Clinic-related factors included clinic volume (annual number of ACLRs/revisions per clinic during the calendar year when the primary ACLR was performed), average clinic volume (median number of ACLRs/revisions per clinic during the three most active years) and the clinic’s ability to use different graft types (HT, PT and QT). The three most active years were chosen for the factor of average clinic volume since some clinics were started or closed during the study period.

### Statistical analysis

The statistical analyses were performed using the SPSS software program (IBM Corp. Released 2017. IBM SPSS Statistics for Macintosh, Version 25.0. Armonk, NY: IBM Corp.). Descriptive statistics were used for patient, surgeon and clinic demographics. Since data were not normally distributed, the median (25th–75th percentile) was reported for continuous variables and the Kruskal–Wallis test was used for between-group comparisons. Categorical variables were reported as the count and percentages, while the χ^2^ test was used for between-groups comparisons. If a significant difference was found, the groups were compared pairwise using the Mann–Whitney U test for continuous variables and the χ^2^ test or Fisher’s exact test for categorical variables. A calculation of Bonferroni-adjusted p values (post hoc correction) was performed for pairwise comparisons.

#### Surgeon-related factors

The surgeon volume, classified as the number of ACLRs/revisions performed by each surgeon per active year, was first assessed as a discrete variable. Like another registry-based study analyzing surgeon and clinic volume in ACLR in the US [13], surgeon volume was then divided into tertiles; low-volume: < 9 ACL surgeries/year, mid-volume: 9–28 surgeries/year and high-volume: > 28 surgeries/year. In the analysis of patient-related data, cases were categorized according to annual surgeon volume during the calendar year when the primary ACLR was performed. In the surgeon-related data, surgeons were assessed separately and classified as low-, mid- or high-volume based on average surgeon volume (median number of ACLRs and revisions during the three most active years in the registry). Surgeons with only two registered years were defined as low-, mid- or high-volume by the median of the two values, while surgeons active for only 1 year in the registry were classified according to the single annual value of ACLRs/revisions. A cut-off for registry caseload of ≥ 50 operations was used, since the number of consecutive procedures to perform an ACLR more accurately and with an improved technique has been suggested as a surgeon caseload of around 50 operations on average [1, 23, 24, 40]. The ability to use different graft types was based on whether the surgeon performed at least one primary ACLR with a certain graft.

#### Clinic-related factors

The clinic volume, defined as the number of ACLRs and revisions per clinic and active year, was processed in the same way as surgeon volume data, leading to the following tertiles: low-volume clinic: < 21 ACL surgeries/year, mid-volume: 21–55 operations/year and high-volume: > 55 operations/year. In the analysis of patient-related data, cases were categorized according to annual clinic volume during the calendar year when the primary ACLR was performed. In the clinic-related data, the low-, mid- and high-volume classification was based on the median number of ACLRs/revisions during the three most active years in the registry per clinic. Regarding the ability of the clinics to use different graft types, the same method was applied as for the surgeons.

#### Factors influencing graft choice

A logistic regression model was used to assess the variables that influence autograft choice during the most recent 5 years, 2015–2019. In the first step, a crude (univariable) analysis was performed for each of the aforementioned variables separately. Patients operated on by surgeons using only one autograft type were excluded here since the surgical treatment, by definition, was standardized rather than individualized. A multivariable logistic regression was then used, beginning with patient-related variables. Injury factors, surgeon-related variables and clinic-related variables were then added in blocks and retained in the model if the p value was < 0.10. The probability of receiving a graft other than HT is presented as the odds ratio (OR) with a 95% confidence interval (CI). The significance level was set at a *p* value of < 0.05.

## Results

**Fig. 1:**
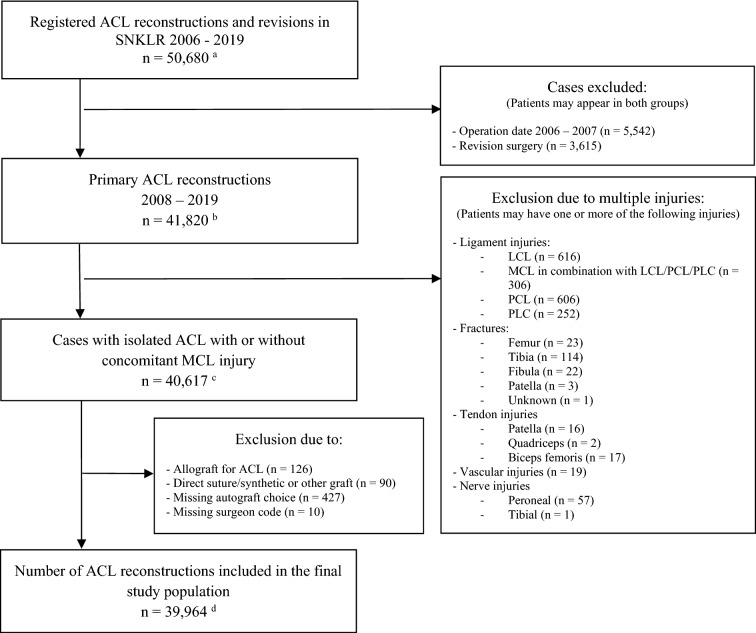
Flowchart of patient selection. *ACL* anterior cruciate ligament, *LCL* lateral collateral ligament, *MCL* medial collateral ligament, *PCL* posterior cruciate ligament, *PLC* posterolateral complex, *SNKLR* Swedish National Knee Ligament Registry. (**a**) *n* Patients = 46,408. (**b**) *n* Patients = 40,288. (**c**) *n* Patients = 39,128. (**d**) *n* Patients = 38,533

**Fig. 2:**
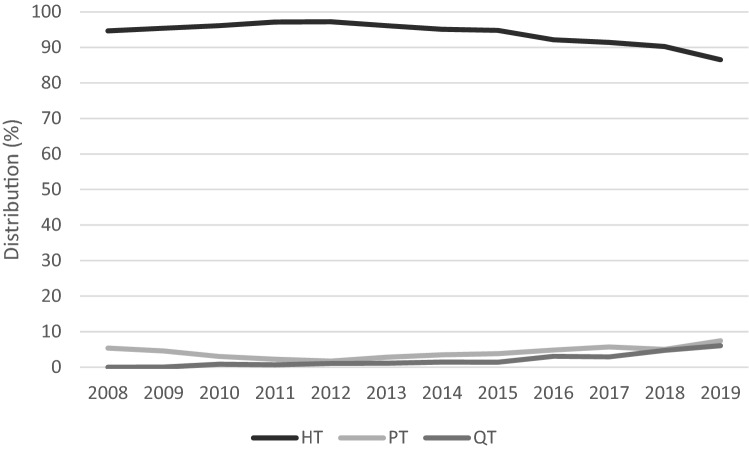
Distribution of graft usage over time for primary ACLR. *ACLR* anterior cruciate ligament reconstruction, *HT* hamstring tendon, *PT* patella tendon, *QT* quadriceps tendon

A total of 39,964 cases (38,533 patients) undergoing primary ACLR with HT, PT or QT autografts between January 1, 2008, and December 31, 2019, were included (Fig. 1). The distribution of graft use over time is presented in Fig. 2. The vast majority of patients received an HT graft (93.7%, *n* = 37,453), whereas PT (4.2%, *n* = 1686) and QT (2.1%, *n* = 825) were used to a lesser extent. The PT graft was used throughout the study period, with a slight decrease in 2008–2012, when the use of HT grafts peaked, but the first registered QT graft was in 2009. The use of PT and QT grafts shows an increasing trend, both reaching the highest levels in 2019 (PT: 7.4%, *n* = 283, and QT: 6.0%, *n* = 229).

### Patient and injury characteristics

Patient and injury characteristics of registered primary ACLRs are presented in Table 1. Almost 44% were females and the median age at surgery was 25. Females and patients younger than 21 years received significantly fewer PT than HT grafts. Football was the leading cause of injury, but regardless of the activity at the time of injury, the HT graft was the primary graft choice in more than 90% of cases; however, patients injured during other pivoting sports received relatively more PT and QT. Only 4.0% had an associated MCL injury as classified at surgery and 18.6% (*n* = 298) of them required surgical treatment. Concomitant MCL injuries were rarely observed among ACLRs performed with HT grafts.

**Table 1:** Demographic data for primary ACLRs 2008–2019 (n = 39,964 cases)

	Total 39,964	HT 37,453	PT 1686	QT 825	*p* value	Test between-groups *p* value, Bonferroni-adjusted		
						HT vs PT	HT vs QT	PT vs QT
Patient sex								
Female	17,529 (43.9)	16,538 (44.2)	645 (38.3)	346 (41.9)	< 0.001	< 0.001	n.s	n.s
Age at surgery^a^								
Median years (25th–75th percentile)	25.0 (19.0–34.0)	25.0 (19.0–34.0)	25.0 (20.0–34.0)	26.0 (20.0–35.0)	< 0.001	0.039	0.005	n.s
< 21 years	13,014 (32.6)	12,273 (32.8)	501 (29.7)	240 (29.1)	0.010	0.042	n.s	n.s
21–30 years	14,177 (35.5)	13,224 (35.3)	645 (38.3)	308 (37.3)				
> 30 years	12,773 (32.0)	11,956 (31.9)	540 (32.0)	277 (33,6)				
Activity at time of injury^a^					< 0.001	0.010	< 0.001	0.003
Football	16,832 (42.1)	15,832 (42.3)	706 (41.9)	294 (35.6)				
Floorball	3392 (8.5)	3227 (8.6)	116 (6.9)	49 (5.9)				
Handball	2168 (5.4)	2029 (5.4)	101 (6.0)	38 (4.6)				
Other pivoting sports^b^	1673 (4.2)	1521 (4.1)	95 (5.6)	57 (6.9)				
Alpine skiing	5804 (14.5)	5.376 (14.4)	256 (15.2)	172 (20.8)				
Other^c^	10,075 (25.2)	9449 (25.2)	411 (24.4)	215 (26.1)				
Associated injury^d^								
Meniscal injury	18,135 (45.4)	16,994 (45.4)	702 (41.6)	439 (53.2)	< 0.001	0.008	< 0.001	< 0.001
Cartilage injury	10,339 (25.9)	9683 (25.9)	392 (23.3)	264 (32.0)	< 0.001	n.s	< 0.001	< 0.001
MCL injury	1606 (4.0)	1186 (3.2)	297 (17.6)	123 (14.9)	< 0.001	< 0.001	< 0.001	n.s
Surgeon volume^e^					< 0.001	< 0.001	< 0.001	< 0.001
Low-volume < 9 cases/year	2315 (5.8)	2159 (5.8)	141(8.4)	15 (1.8)				
Mid-volume 9–28 cases/year	10,443 (26.1)	9958 (26.6)	411 (24.4)	74 (9.0)				
High-volume > 28 cases/year	27,206 (68.1)	25,336 (67.6)	1134 (67.3)	736 (89.2)				
Surgeon caseload								
≥ 50 ACLRs^f^	34,010 (85.1)	31,766 (84.8)	1437 (85.2)	807 (97.8)	< 0.001	n.s	< 0.001	< 0.001
Surgeon graft use^g^					< 0.001	< 0.001	< 0.001	< 0.001
1 graft	5627 (14.1)	5506 (14.7)	121 (7.2)	0 (0.0)				
2 grafts	22,627 (56.6)	21,147 (56.5)	1069 (63.9)	411 (49.8)				
3 grafts	11,710 (29.3)	10,800 (28.8)	496 (29.4)	414 (50.2)				
Clinic volume^h^					< 0.001	< 0.001	< 0.001	< 0.001
Low-volume < 21 cases/year	2668 (6.7)	2487 (6.6)	166 (9.8)	15 (1.8)				
Mid-volume 21–55 cases/year	8444 (21.1)	8032 (21.4)	257 (15.2)	155 (18.8)				
High-volume > 55 cases/year	28,852 (72.2)	26,934 (71.9)	1263 (74.9)	655 (79.4)				
Clinic graft use^i^					< 0.001	< 0.001	< 0.001	< 0.001
1 graft	2787 (7.0)	2712 (7.2)	75 (4.4)	0				
2 grafts	18,087 (45.3)	17,187 (45.9)	752 (44.6)	148 (17.9)				
3 grafts	19,090 (47.8)	17,554 (46.9)	859 (50.9)	677 (82.1)				

Data are reported as *n* (%), unless otherwise indicated

*ACLR* anterior cruciate ligament reconstruction, *HT* hamstring tendon, *MCL* medial collateral ligament, *n.s.* not significant, *PT* patellar tendon, *QT* quadriceps tendon

^a^Missing values: age at surgery—40 patients missing; activity at time of injury—20 patients missing

^b^Other pivoting sport = basketball, hockey, rugby and American football

^c^Other = other sports, exercise, work, traffic accident and other reasons

^d^Each patient may present with one or more of the associated injuries

^e^Yearly surgeon volume when primary ACLR was performed

^f^Primary ACLR performed by a surgeon with at least 50 previous ACLRs or revisions

^g^Primary ACLR performed by a surgeon that has used 1–3 autograft types

^h^Yearly clinic volume when primary ACLR was performed

^i^Primary ACLR performed at a clinic that has used 1–3 autograft types

### Surgeon-related factors

The primary ACLRs were performed by 299 surgeons with a registry caseload from 1 to 1106 ACLRs/revisions. Baseline patient data regarding surgeon-related factors are presented in Table 1. More than two-thirds of patients were operated on by high-volume surgeons (> 28 cases/year) and the majority by surgeons with a total registry caseload of at least 50 ACLRs/revisions (85.1%) and surgeons able to perform primary ACLRs with at least two graft types (85.9%). Moreover, primary ACLR with a QT graft was almost solely performed by high-volume surgeons, surgeons with a caseload of ≥ 50 ACL surgeries and in all cases by a surgeon that used at least two graft types. Even so, high-volume surgeons used HT grafts in 93.1% of all their primary ACLRs.

The median yearly surgical volume per surgeon during the three most active years was 16 (5.5–40) surgeries. Surgeon characteristics are presented in Table 2. Nearly all the surgeons were able to perform primary ACLRs with HT grafts and slightly more than half used PT grafts, whereas only one-sixth performed ACLRs with QT grafts. Almost half the surgeons performed ACLRs with only one graft type.

**Table 2:** Surgeon characteristics for registered ACLRs 2008–2019 (*n* = 299 surgeons)

	Total 299	HT 290	PT 159	QT 48	*p* value	Test between-groups *p* value, Bonferroni-adjusted		
						HT vs PT	HT vs QT	PT vs QT
Average yearly volume^a^					< 0.001	< 0.001	< 0.001	n.s
Low-volume < 9 cases/year	100 (33.4)	94 (32.4)	16 (10.1)	2 (4.2)				
Mid-volume 9–28 cases/year	91 (30.4)	88 (30.8)	50 (31.4)	11 (22.9)				
High-volume > 28 cases/year	108 (36.1)	108 (37.2)	93 (58.5)	35 (72.9)				
Caseload								
≥ 50 ACLR^b^	159 (53.2)	158 (54.5)	112 (70.4)	41 (85.4)	< 0.001	0.003	< 0.001	n.s
No. of used graft types^c^					< 0.001	< 0.001	< 0.001	< 0.001
1 graft	140 (46.8)	131 (45.2)	9 (5.7)	0 (0.0)				
2 grafts	120 (40.1)	120 (41.4)	111 (68.9)	9 (18.8)				
3 grafts	39 (13.0)	39 (13.4)	39 (24.5)	39 (81.3)				

Data are reported as *n* (%). Each surgeon may appear in one or several groups (HT, PT, QT)

*ACLR* anterior cruciate ligament reconstruction, *HT* hamstring tendon, *n.s.* not significant, *PT* patellar tendon, *QT* quadriceps tendon

^a^Average yearly volume expressed as the median of the three most active years per surgeon

^b^Surgeon has performed at least 50 previous ACLRs or revisions

^c^Surgeon has used 1–3 autograft types in primary ACLRs

### Clinic-related factors

The ACL surgeries were performed at 91 different clinics. Demographic patient data regarding clinic-related factors are presented in Table 1. Most patients underwent primary ACLR at a high-volume clinic (72.2%) and underwent surgery at a clinic capable of using at least two autograft types (93.1%). The majority of patients reconstructed at high-volume clinics received an HT autograft (93.3%).

The median surgical volume per clinic during the three most active years was 34 (16–69) surgeries. Clinic characteristics are shown in Table 3. Primary ACLR with an HT graft was performed at all but two clinics. Every fourth clinic performed primary ACL reconstructions with only one graft type.

**Table 3:** Clinic characteristics of registered ACLRs 2008–2019 (*n* = 91 clinics)

	Total 91	HT 89	PT 65	QT 23	*p* value	Test between-groups *p* value, Bonferroni-adjusted		
						HT vs PT	HT vs QT	PT vs QT
Average yearly volume^a^					n.s			
Low-volume < 21 cases/year	30 (33.0)	28 (31.5)	17 (26.2)	4 (17.4)				
Mid-volume 21–55 cases/year	32 (35.2)	32 (32.6)	22 (33.8)	6 (26.1)				
High-volume > 55 cases/year	29 (31.2)	29 (32.6)	26 (40.0)	13 (56.5)				
No. of used graft types^b^					< 0.001	0.009	< 0.001	< 0.001
1 graft	22 (24.2)	20 (22.5)	2 (3.1)	0 (0.0)				
2 grafts	52 (57.1)	52 (58.4)	46 (70.8)	6 (26.1)				
3 grafts	17 (18.7)	17 (19.1)	17 (26.5)	17 (73.9)				

Data are reported as *n* (%). Each clinic may appear in one or several groups (HT, PT, QT)

*ACLR* anterior cruciate ligament reconstruction, *HT* hamstring tendon, *n.s.* not significant, *PT* patellar tendon, *QT* quadriceps tendon

^a^Average yearly volume expressed as the median of the three most active years per clinic

^b^Clinic has used 1–3 autograft types in primary ACLRs

### Factors influencing graft choice

Factors influencing the probability of receiving a graft other than an HT are presented in Table 4. Significantly increased odds of receiving a PT or QT graft were found for ACLR by surgeons with a caseload of ≥ 50 in the univariable logistic regression, as well as injury during handball and other pivoting sports, a concomitant MCL injury and later year of surgery. On the other hand, injury during floorball, ACLR by low- and mid-volume surgeons and surgery at low- and mid-volume clinics were found to have significantly reduced odds of receiving PT or QT grafts. Age and participation in football and alpine skiing did not influence graft selection.

**Table 4:** Factors influencing probability of receiving graft (PT/QT) other than HT in primary ACLR 2015–2019

Variable	Crude (univariable)		Adjusted (multivariable)^a^	
	OR (95% CI)	*p* value	OR (95% CI)	*p* value
Patient factors				
Female	0.907 (0.817–1.006)	n.s	0.866 (0.774–0.969)	0.012
Age at surgery				
< 21 years	1.023 (0.898–1.165)	n.s	1.058 (0.914–1.223)	n.s
21–30 years	1.098 (0.971–1.243)	n.s	1.137 (0.995–1.300)	n.s
> 30 years	Ref		Ref	
Injury factors				
Activity at time of injury				
Football	0.998 (0.874–1.140)	n.s	1.022 (0.885–1.180)	n.s
Floorball	0.666 (0.523–0.849)	0.001	0.710 (0.553–0.911)	0.007
Handball	1.275 (1.010–1.609)	0.041	1.305 (1.021–1.669)	0.034
Other pivoting sports^b^	1.685 (1.335–2.128)	< 0.001	1.590 (1.244–2.033)	< 0.001
Alpine skiing	1.145 (0.977–1.342)	n.s	1.063 (0.901–1.254)	n.s
Other	Ref		Ref	
Associated injury				
Meniscal injury	0.968 (0.874–1.074)	n.s	n.s	
Cartilage injury	0.951 (0.845–1.070)	n.s	n.s	
MCL injury	4.996 (4.261–5.858)	< 0.001	4.926 (4.181–5.804)	< 0.001
Year of surgery	1.256 (1.209–1.305)	< 0.001	1.255 (1.207–1.306)	< 0.001
Surgeon factors				
Yearly volume				
Low-volume < 9 cases/year	0.683 (0.481–0.969)	0.033	0.861 (0.586–1.265)	n.s
Mid-volume 9–28 cases/year	0.581 (0.505–0.667)	< 0.001	0.623 (0.533–0.727)	< 0.001
High-volume > 28 cases/year	Ref		Ref	
Caseload ≥ 50 ACLRs	1.751 (1.388–2.209)	< 0.001	1.406 (1.105–1.789)	0.006
Clinic factors				
Yearly volume				
Low-volume < 21 cases/year	0.501 (0.381–0.658)	< 0.001	0.749 (0.552–1.015)	n.s
Mid-volume 21–55 cases/year	0.806 (0.695–0.935)	< 0.001	1.075 (0.916–1.262)	n.s
High-volume > 55 cases/year	Ref		Ref	

Data are presented as the odds ratio (95% confidence interval)

*ACLR* anterior cruciate ligament reconstruction, *CI* confidence interval, *HT* hamstring tendon, *MCL* medial collateral ligament, *n.s.* not significant, *OR* odds ratio, *PT* patellar tendon, *QT* quadriceps tendon, *ref* reference

^a^Adjusted for all included variables

^b^Other pivoting sport = basketball, hockey, rugby and American football

^c^Other = other sports, exercise, work, traffic accident and other reasons

There were 14,259 HT cases and 1617 PT/QT cases remaining in the multivariable analysis. Females had a reduced probability of receiving PT or QT grafts, whereas there were no differences between low- and high-volume surgeons and between low-, mid-, and high-volume clinics. Otherwise, the same main results were achieved in both the multivariable and the univariable analysis.

## Discussion

The principal finding in this study, which is the first from the SNKLR to broadly assess surgeon experience and clinic volume, was that patients received relatively more PT and QT autografts if they were operated on by more experienced surgeons. Additionally, injury during participation in various pivoting sports and a concomitant MCL injury at the time of ACLR were also found to influence graft selection in favor of PT or QT autografts.

### Graft choice

Previous registry-based studies confirm the use of HT as the most common autograft choice [25, 32, 33]. Additionally, studies investigating surgeon preference found a predilection for HT autografts in primary ACLR [14, 52]. However, there is an emerging trend toward increased PT and QT graft utilization in Scandinavia [46, 47]. It is therefore important that surgeons understand the risks and benefits of available graft options and are capable of using several grafts to meet the individual needs of each patient.

ACLR with an HT autograft is associated with lower donor-site morbidity [17, 19, 54], but with an increased risk of septic arthritis [15] and weakening of deep knee flexion strength [38] compared with a PT autograft. A PT autograft results in an objectively more stable knee, albeit with an increased risk of anterior knee pain [6, 19, 54] and the development of osteoarthritis [17, 50]. Some studies have also found higher return to preinjury level sport frequencies [7, 54] and a reduced risk of re-rupture and revision in favor of the PT autograft compared with the HT autograft [7, 8, 20, 27, 28, 37]. However, both HT and PT autografts yield high subjective knee function [20], but considering that the revision rate associated with HT autograft is up to 4 times higher than with the PT autograft, it is questionable whether the “one-fits-all” use of HT as the primary choice is evidence-based [8, 27, 28, 31, 33, 34]. These recent findings have likely influenced Norwegian ACL surgeons, leading to a reduction in HT autograft use in Norway from 84% in 2010 to 28% in 2019 and an increase in PT autograft use from 16 to 72% respectively [31, 47]. In this study, ACLRs with HT autografts have declined in recent years, but they are still used frequently (86.5% in 2019). Since 2015, there has, however, been a significant annual increase in the use of PT or QT autografts, illustrating a change in graft preference even among Swedish ACL surgeons.

The role of the QT autograft in primary ACLR is less well studied. Nonetheless, the QT autograft has been found to have a similar or lower rate of donor-site morbidity compared with the HT autograft [11, 22]. At the same time, QT harvesting is associated with a reduction in quadriceps strength [22]. Further comparisons of the QT autograft have revealed similar or improved knee stability, return to activity level, graft survival rate and subjective outcome scores compared with the HT autograft [11, 22, 29]. Additionally, studies comparing the QT and PT autografts report a lower rate of donor-site morbidity in favor of QT but no difference in knee stability, risk of re-rupture and subjective knee function [29]. These potentially advantageous findings have probably contributed to the slow, but steady increase in QT utilization seen in Sweden in this study. A trend of this kind has also been identified recently in Denmark, where QT autograft use increased from 2% in 2012 to 11% in 2019 [15].

### Patient and injury factors

The graft choice based on patient sex is in agreement with previous reports from national ACL registries and surgeon surveys [21, 33, 52], but the underlying reasons are still somewhat unclear. While female sex has been identified as a risk factor for primary ACL injury, there is conflicting evidence regarding the risk of graft rupture and revision rates between the sexes [3, 5, 25, 43, 45]. In terms of graft characteristics, a few studies have found an increase in objective knee stability and a lower incidence of graft rupture in primary ACLRs with PT compared with HT autografts among young females [35, 39]. It is therefore debatable whether the significant difference in graft distribution between the sexes, seen in this study, is evidence-based and females perhaps receive less individualized treatment compared with males.

Interestingly, patients injured during handball and other pivoting sports, such as basketball, hockey, rugby and American football (but not football and floorball), had a higher probability of receiving PT or QT autografts. One possible explanation is that football and floorball are widely practiced at all the different non-elite recreational levels in Sweden, while handball and the other pivoting sports have fewer practitioners and thus have a higher ratio of highly active athletes. Although, level of activity was not assessed, thereby limiting the ability to draw conclusions.

Finally, patients with a concomitant MCL injury at the ACLR had five times higher odds of receiving PT or QT autografts. The HT contributes to the valgus stability in knee extension and it has therefore been suggested that it plays an important role in preserving the ipsilateral HT in MCL-insufficient knee joints [10]. However, even if non-surgically treated, concomitant MCL injuries at ACLR have been found possibly to increase the risk of revision surgery [42], the use of HT autografts has not been linked to an increased risk in comparison to PT [44]. According to our results, Swedish ACL surgeons appear to adjust their graft choice in the presence of a concomitant MCL injury, but further studies are needed to evaluate whether the individualization is evidence-based.

### Surgeon experience and clinic volume

Many surgeons perform a limited number of ACLRs annually, but most patients are operated on by experienced surgeons and at high-volume clinics. This finding is consistent with reports from other countries, such as the UK, the US and the Netherlands [14, 47, 49]. However, the literature on surgeon experience and clinic volume and their association with graft choice in primary ACLR is scarce. In our study, the ability to use different graft types and the tendency toward individualization increased with surgeon experience. High-volume surgeons and surgeons with a registry caseload of at least 50 primary ACLRs/revisions were more likely to use PT or QT autografts, whereas clinic volume did not influence graft selection. Moreover, a primary ACLR with a QT graft was almost solely performed by experienced surgeons. A recently published study found that high-volume surgeons (> 25 ACLRs/year) and clinics (> 125 ACLRs/year) in the US were more likely to use autografts than allografts [18]. Another US study analyzed data from the Kaiser Permanente ACLR Registry between 2005 and 2010 and found that high-volume (≥ 52 ACLRs/year) and fellowship-trained surgeons preferred PT and then HT autografts [12]. Taken as a whole, these findings indicate that surgeon experience is associated with different graft utilization and possibly with treatment individualization. It is important to emphasize this in the clinical setting, as previous research has repeatedly demonstrated significant differences in graft characteristics. When considering whether graft individualization is important and to optimize the treatment given to each unique patient, it is mandatory for surgeons to be able to use more than one graft option. The findings in this study suggest that patients might benefit from surgery being performed by high-volume surgeons, as PT/QT autografts were used relatively more frequently by experienced surgeons. Surgeon experience might be an important factor to adjust for in multiple-surgeon studies of graft choice in primary ACLR. However, it also remains to be investigated whether graft individualization and surgeon experience influence outcomes, such as the risk of re-rupture and results relating to patient-related outcome measurements.

Even though an HT autograft was used in more than 90% of all ACLRs during the study period, the large sample of almost 40,000 ACLRs also enabled us to analyze both PT and QT autografts, as well as surgeon experience and clinic volume. However, some innate limitations are associated with registry-based studies like ours, since they rely on both patients and surgeons to report and enter data correctly. For example, non-surgically treated MCL injuries are probably underreported in the SNKLR and the findings relating to MCL injuries should therefore be interpreted with some caution. Additionally, the non-exclusion of younger patients with open growth zones might have masked a potential difference in graft selection based on patient age. Next, another limitation is the lack of certain data, such as patient activity level, which prevented further analyses of graft individualization. In this study, activity at the time of injury was used as a general indication of patients’ activity level, but it does not reveal whether a person is moderately active or competing at elite level. It is therefore important to assess activity level in future studies, since this could influence the graft choice decision. Finally, the definition of surgeon experience and clinic routine varies across the literature and there are no established cut-off values defining low- to high-volume surgeons and clinics.

## Conclusion

Patients operated on by more experienced surgeons received relatively more PT and QT autografts, whereas clinic volume did not appear to influence graft selection. An increase in the use of PT/QT autografts was also seen among patients injured during some pivoting sports and patients presenting with a concomitant MCL injury at ACLR. Even though most Swedish patients were operated on by experienced surgeons and at high-volume clinics, the majority still received HT autografts.

## Abbreviations

ACL: Anterior cruciate ligament
ACLR: Anterior cruciate ligament reconstruction
CI: Confidence interval
HT: Hamstring tendon
LCL: Lateral collateral ligament
MCL: Medial collateral ligament
OR: Odds ratio
PCL: Posterior cruciate ligament
PLC: Posterolateral complex
PT: Patella tendon
QT: Quadriceps tendon
SNKLR: Swedish National Knee Ligament Registry

## References

[1] Behrend H, Stutz G, Kessler MA, Rukavina A, Giesinger K, Kuster MS (2006). Tunnel placement in anterior cruciate ligament (ACL) reconstruction: quality control in a teaching hospital. Knee Surg Sports Traumatol Arthrosc.

[2] Bowman EN, Limpisvasti O, Cole BJ, ElAttrache NS (2021). Anterior cruciate ligament reconstruction graft preference most dependent on patient age: a survey of United States surgeons. Arthroscopy.

[3] Capogna BM, Mahure SA, Mollon B, Duenes ML, Rokito AS (2020). Young age, female gender, Caucasian race, and workers’ compensation claim are risk factors for reoperation following arthroscopic ACL reconstruction. Knee Surg Sports Traumatol Arthrosc.

[4] Chechik O, Amar E, Khashan M, Lador R, Eyal G, Gold A (2013). An international survey on anterior cruciate ligament reconstruction practices. Int Orthop.

[5] Cristiani R, Forssblad M, Edman G, Eriksson K, Stålman A (2021). Age, time from injury to surgery and quadriceps strength affect the risk of revision surgery after primary ACL reconstruction. Knee Surg Sports Traumatol Arthrosc.

[6] Cristiani R, Sarakatsianos V, Engström B, Samuelsson K, Forssblad M, Stålman A (2019). Increased knee laxity with hamstring tendon autograft compared to patellar tendon autograft: a cohort study of 5462 patients with primary anterior cruciate ligament reconstruction. Knee Surg Sports Traumatol Arthrosc.

[7] DeFazio MW, Curry EJ, Gustin MJ, Sing DC, Abdul-Rassoul H, Ma R, Fu F, Li X (2020). Return to sport after ACL reconstruction with a BTB versus hamstring tendon autograft: a systematic review and meta-analysis. Orthop J Sports Med.

[8] Ekeland A, Engebretsen L, Fenstad AM, Heir S (2020). Similar risk of ACL graft revision for alpine skiers, football and handball players: the graft revision rate is influenced by age and graft choice. Br J Sports Med.

[9] Filbay SR, Grindem H (2019). Evidence-based recommendations for the management of anterior cruciate ligament (ACL) rupture. Best Pract Res Clin Rheumatol.

[10] Herbort M, Michel P, Raschke MJ, Vogel N, Schulze M, Zoll A, Fink C, Petersen W, Domnick C (2017). Should the ipsilateral hamstrings be used for anterior cruciate ligament reconstruction in the case of medial collateral ligament insufficiency? Biomechanical investigation regarding dynamic stabilization of the medial compartment by the hamstring muscles. Am J Sports Med.

[11] Hurley ET, Calvo-Gurry M, Withers D, Farrington SK, Moran R, Moran CJ (2018). Quadriceps tendon autograft in anterior cruciate ligament reconstruction: a systematic review. Arthroscopy.

[12] Inacio MCS, Paxton EW, Maletis GB, Csintalan RP, Granan L-P, Fithian DC, Funahashi TT (2012). Patient and surgeon characteristics associated with primary anterior cruciate ligament reconstruction graft selection. Am J Sports Med.

[13] Jain N, Pietrobon R, Guller U, Shankar A, Ahluwalia AS, Higgins LD (2005). Effect of provider volume on resource utilization for surgical procedures of the knee. Knee Surg Sports Traumatol Arthrosc.

[14] Koc BB, Schotanus MGM, Jansen EJP (2021). Preferences in anterior cruciate ligament reconstruction and return to sport: a survey among surgeons in the Netherlands. J Clin Orthop Trauma.

[15] Kraus Schmitz J, Lindgren V, Edman G, Janarv P-M, Forssblad M, Stålman A (2021). Risk factors for septic arthritis after anterior cruciate ligament reconstruction: a nationwide analysis of 26,014 ACL reconstructions. Am J Sports Med.

[16] Kurz A, Evaniew N, Yeung M, Samuelsson K, Peterson D, Ayeni OR (2017). Credibility and quality of meta-analyses addressing graft choice in anterior cruciate ligament reconstruction: a systematic review. Knee Surg Sports Traumatol Arthrosc.

[17] Leys T, Salmon L, Waller A, Linklater J, Pinczewski L (2012). Clinical results and risk factors for reinjury 15 years after anterior cruciate ligament reconstruction: a prospective study of hamstring and patellar tendon grafts. Am J Sports Med.

[18] Li LT, Bokshan SL, DeFroda SF, Mehta SR, Fadale PD, Owens BD (2020). High case volume predicts greater odds of autograft use and meniscal repair for anterior cruciate ligament reconstruction. Arthroscopy.

[19] Li S, Chen Y, Lin Z, Cui W, Zhao J, Su W (2012). A systematic review of randomized controlled clinical trials comparing hamstring autografts versus bone-patellar tendon-bone autografts for the reconstruction of the anterior cruciate ligament. Arch Orthop Trauma Surg.

[20] Lin KM, Boyle C, Marom N, Marx RG (2020). Graft selection in anterior cruciate ligament reconstruction. Sports Med Arthrosc Rev.

[21] Lind M, Strauss MJ, Nielsen T, Engebretsen L (2021). Low surgical routine increases revision rates after quadriceps tendon autograft for anterior cruciate ligament reconstruction: results from the Danish knee ligament reconstruction registry. Knee Surg Sports Traumatol Arthrosc.

[22] Lind M, Nielsen TG, Soerensen OG, Mygind-Klavsen B, Faunø P (2020). Quadriceps tendon grafts does not cause patients to have inferior subjective outcome after anterior cruciate ligament (ACL) reconstruction than do hamstring grafts: a 2-year prospective randomised controlled trial. Br J Sports Med.

[23] Luthringer TA, Blackmore SA, Singh BC, Strauss EJ (2016). The learning curve associated with anteromedial portal drilling in ACL reconstruction. Phys Sportsmed.

[24] Lyman S, Koulouvaris P, Sherman S, Do H, Mandl LA, Marx RG (2009). Epidemiology of anterior cruciate ligament reconstruction: trends, readmissions, and subsequent knee surgery. J Bone Joint Surg Am.

[25] Maletis GB, Funahashi TT, Inacio MCS, Paxton LW (2022). Optimizing anterior cruciate ligament reconstruction: individualizing the decision-making process using data from the Kaiser Permanente ACLR registry: 2018 OREF award paper. J Orthop Res.

[26] Mall NA, Chalmers PN, Moric M, Tanaka MJ, Cole BJ, Bach BR, Paletta GA (2014). Incidence and trends of anterior cruciate ligament reconstruction in the United States. Am J Sports Med.

[27] Mohtadi NG, Chan DS (2019). A randomized clinical trial comparing patellar tendon, hamstring tendon, and double-bundle ACL reconstructions: patient-reported and clinical outcomes at 5-year follow-up. J Bone Joint Surg Am.

[28] Spindler KP, Huston LJ, Zajichek A, Reinke EK, Amendola A, Andrish JT, Brophy RH, Dunn WR, Flanigan DC, Jones MH, Kaeding CC, Marx RG, Matava MJ, McCarty EC, Parker RD, Vidal AF, Wolcott ML, Wolf BR, Wright RW, MOON Knee Group (2020). Anterior cruciate ligament reconstruction in high school and college-aged athletes: does autograft choice influence anterior cruciate ligament revision rates?. Am J Sports Med.

[29] Mouarbes D, Menetrey J, Marot V, Courtot L, Berard E, Cavaignac E (2019). Anterior cruciate ligament reconstruction: a systematic review and meta-analysis of outcomes for quadriceps tendon autograft versus bone–patellar tendon–bone and hamstring-tendon autografts. Am J Sports Med.

[30] Nordenvall R, Bahmanyar S, Adami J, Stenros C, Wredmark T, Felländer-Tsai L (2012). A population-based nationwide study of cruciate ligament injury in Sweden, 2001–2009: incidence, treatment, and sex differences. Am J Sports Med.

[31] Persson A, Fjeldsgaard K, Gjertsen J-E, Kjellsen AB, Engebretsen L, Hole RM, Fevang JM (2014). Increased risk of revision with hamstring tendon grafts compared with patellar tendon grafts after anterior cruciate ligament reconstruction: a study of 12,643 patients from the Norwegian Cruciate Ligament Registry, 2004–2012. Am J Sports Med.

[32] Prentice HA, Lind M, Mouton C, Persson A, Magnusson H, Gabr A, Seil R, Engebretsen L, Samuelsson K, Karlsson J, Forssblad M, Haddad FS, Spalding T, Funahashi TT, Paxton LW, Maletis GB (2018). Patient demographic and surgical characteristics in anterior cruciate ligament reconstruction: a description of registries from six countries. Br J Sports Med.

[33] Rahardja R, Zhu M, Love H, Clatworthy MG, Monk AP, Young SW (2020). Effect of graft choice on revision and contralateral anterior cruciate ligament reconstruction: results from the New Zealand ACL registry. Am J Sports Med.

[34] Rahr-Wagner L, Thillemann TM, Pedersen AB, Lind M (2014). Comparison of hamstring tendon and patellar tendon grafts in anterior cruciate ligament reconstruction in a nationwide population-based cohort study: results from the Danish registry of knee ligament reconstruction. Am J Sports Med.

[35] Salem HS, Varzhapetyan V, Patel N, Dodson CC, Tjoumakaris FP, Freedman KB (2019). Anterior cruciate ligament reconstruction in young female athletes: patellar versus hamstring tendon autografts. Am J Sports Med.

[36] Salminen M, Kraeutler MJ, Freedman KB, Tucker BS, Salvo JP, Ciccotti MG, Cohen SB (2016). Choosing a graft for anterior cruciate ligament reconstruction: surgeon influence reigns supreme. Am J Orthop.

[37] Samuelsen BT, Webster KE, Johnson NR, Hewett TE, Krych AJ (2017). Hamstring autograft versus patellar tendon autograft for ACL reconstruction: is there a difference in graft failure rate? A meta-analysis of 47,613 patients. Clin Orthop Relat Res.

[38] Samuelsson K, Andersson D, Karlsson J (2009). Treatment of anterior cruciate ligament injuries with special reference to graft type and surgical technique: an assessment of randomized controlled trials. Arthroscopy.

[39] Shakked R, Weinberg M, Capo J, Jazrawi L, Strauss E (2016). Autograft choice in young female patients: patella tendon versus hamstring. J Knee Surg.

[40] Sirleo L, Innocenti M, Innocenti M, Civinini R, Carulli C, Matassi F (2018). Post-operative 3D CT feedback improves accuracy and precision in the learning curve of anatomic ACL femoral tunnel placement. Knee Surg Sports Traumatol Arthrosc.

[41] Slone HS, Romine SE, Premkumar A, Xerogeanes JW (2015). Quadriceps tendon autograft for anterior cruciate ligament reconstruction: a comprehensive review of current literature and systematic review of clinical results. Arthroscopy.

[42] Svantesson E, Hamrin Senorski E, Alentorn-Geli E, Westin O, Sundemo D, Grassi A, Čustović S, Samuelsson K (2019). Increased risk of ACL revision with non-surgical treatment of a concomitant medial collateral ligament injury: a study on 19,457 patients from the Swedish National Knee Ligament Registry. Knee Surg Sports Traumatol Arthrosc.

[43] Svantesson E, Hamrin Senorski E, Baldari A, Ayeni OR, Engebretsen L, Franceschi F, Karlsson J, Samuelsson K (2019). Factors associated with additional anterior cruciate ligament reconstruction and register comparison: a systematic review on the Scandinavian knee ligament registers. Br J Sports Med.

[44] Svantesson E, Hamrin Senorski E, Östergaard M, Grassi A, Krupic F, Westin O, Samuelsson K (2020). Graft choice for anterior cruciate ligament reconstruction with a concomitant non-surgically treated medial collateral ligament injury does not influence the risk of revision. Arthroscopy.

[45] Tan SHS, Lau BPH, Khin LW, Lingaraj K (2016). The importance of patient sex in the outcomes of anterior cruciate ligament reconstructions: a systematic review and meta-analysis. Am J Sports Med.

[46] The Danish Knee Ligament Reconstruction Registry. Annual Report 2020/2021. https://www.sundhed.dk/content/cms/0/4700_dkrr_aarsrapport_2020_offentliggorelse.pdf. Accessed 18 Jan. 2022.

[47] The Norwegian Knee Ligament Registry. Annual Report 2019. https://www.kvalitetsregistre.no/register/muskel-og-skjelett/nasjonalt-korsbandregister. Accessed 18 Jan. 2022.

[48] The Swedish National Knee Ligament Registry. Annual Report 2019. http://www.aclregister.nu. Accessed 18 Jan. 2022.

[49] The UK National Ligament Registry. Annual Report 2020. https://www.uknlr.co.uk/media.php. Accessed 18 Jan. 2022.

[50] Thompson SM, Salmon LJ, Waller A, Linklater J, Roe JP, Pinczewski LA (2016). Twenty-year outcome of a longitudinal prospective evaluation of isolated endoscopic anterior cruciate ligament reconstruction with patellar tendon or hamstring autograft. Am J Sports Med.

[51] Tibor L, Chan PH, Funahashi TT, Wyatt R, Maletis GB (2014). Inacio MCS (2016) Surgical technique trends in primary ACL reconstruction from 2007 to. J Bone Joint Surg Am.

[52] Vascellari A, Grassi A, Canata GL, Zaffagnini S, Gokeler A, Jones H (2021). Hamstrings substitution via anteromedial portal with optional anterolateral ligament reconstruction is the preferred surgical technique for anterior cruciate ligament reconstruction: a survey among ESSKA members. Knee Surg Sports Traumatol Arthrosc.

[53] Widner M, Dunleavy M, Lynch S (2019). Outcomes following ACL reconstruction based on graft type: are all grafts equivalent?. Curr Rev Musculoskelet Med.

[54] Xie X, Liu X, Chen Z, Yu Y, Peng S, Li Q (2015). A meta-analysis of bone–patellar tendon–bone autograft versus four-strand hamstring tendon autograft for anterior cruciate ligament reconstruction. Knee.

